# Physical Weight Loading Induces Expression of Tryptophan Hydroxylase 2 in the Brain Stem

**DOI:** 10.1371/journal.pone.0085095

**Published:** 2014-01-08

**Authors:** Joon W. Shim, Todd R. Dodge, Max A. Hammond, Joseph M. Wallace, Feng C. Zhou, Hiroki Yokota

**Affiliations:** 1 Department of Biomedical Engineering, Indiana University - Purdue University Indianapolis, Indianapolis, Indiana, United States of America; 2 Weldon School of Biomedical Engineering, Purdue University, West Lafayette, Indiana, United States of America; 3 Department of Orthopaedic Surgery, Indiana University School of Medicine, Indianapolis, Indiana, United States of America; 4 Department of Anatomy and Cell Biology, Indiana University School of Medicine, Indianapolis, Indiana, United States of America; 5 Stark Neuroscience Research Institute, Indiana University School of Medicine, Indianapolis, Indiana, United States of America; National Institute of Genomic Medicine, Mexico

## Abstract

Sustaining brain serotonin is essential in mental health. Physical activities can attenuate mental problems by enhancing serotonin signaling. However, such activity is not always possible in disabled individuals or patients with dementia. Knee loading, a form of physical activity, has been found to mimic effects of voluntary exercise. Focusing on serotonergic signaling, we addressed a question: Does local mechanical loading to the skeleton elevate expression of tryptophan hydroxylase 2 (tph2) that is a rate-limiting enzyme for brain serotonin? A 5 min knee loading was applied to mice using 1 N force at 5 Hz for 1,500 cycles. A 5-min treadmill running was used as an exercise (positive) control, and a 90-min tail suspension was used as a stress (negative) control. Expression of tph2 was determined 30 min – 2 h in three brain regions ––frontal cortex (FC), ventromedial hypothalamus (VMH), and brain stem (BS). We demonstrated for the first time that knee loading and treadmill exercise upregulated the mRNA level of tph2 in the BS, while tail suspension downregulated it. The protein level of tph2 in the BS was also upregulated by knee loading and downregulated by tail suspension. Furthermore, the downregulation of tph2 mRNA by tail suspension can be partially suppressed by pre-application of knee loading. The expression of tph2 in the FC and VMH was not significantly altered with knee loading. In this study we provided evidence that peripheral mechanical loading can activate central tph2 expression, suggesting that physical cues may mediate tph2-cathalyzed serotonergic signaling in the brain.

## Introduction

Mood disorder is a commonly encountered health problem that could lead to anxiety, depression, and in some cases, suicide. Physical activities are commonly recommended as a preventive measure because of their stimulatory role in pain reduction, formation of new neurons as well as synthesis of neurotransmitters [1], [2], [3]. However, routine exercises are not always possible, in particular, for the elderly and physically disabled individuals. Since availability of serotonin in the brain is thought to be key to mood disorder, we aimed to develop a therapeutic exercise regimen focusing on mechanical loading to the skeleton. Knee loading is a recently developed mechanical loading modality, which applies lateral loads to the knee to induce anabolic responses of the skeleton [4], [5]. It stimulates bone formation, and accelerates bone wound healing in the lower limb [6], [7]. A unique feature of knee loading is its extended effects not only to the loaded but also to non-loaded contralateral bone [5]. Little is known, however, about its effect on the brain.

In this study, we addressed a question: Does mechanical loading to the knee elevate serotonin signaling in the brain? Serotonin in the brain is known to elevate by physical activities [8]. Although bone remodeling is influenced by serotonin [9], [10], [11], effect of knee loading on serotonin in the brain has not ever been reported. Since skeletal loading is a significant element of physical activities, we hypothesized that gentle cyclic loading to the joint stimulates expression of tryptophan hydroxylase 2 (tph2), which is the rate-limiting enzyme for serotonin in the brain.

In order to test the hypothesis, we employed treadmill exercises as a positive control, while unloading by tail suspension, which mimics disuse-induced stress or syndrome, as a negative control. Knee loading was applied by a custom-made piezoelectric loader [5], [6], [12] and gene expression in brain tissues such as the frontal cortex (FC), ventromedial hypothalamus (VMH), and raphe nuclei of brain stem (BS) was examined. The FC is engaged in cognitive and motor responses that are sensitive to serotonin signaling [13], [14], while the VMH is linked to feeding, thermoregulation, and sexual activity [15]. The raphe nuclei of BS is a site of serotonin synthesis, whose rate-limiting enzymatic reaction is catalyzed by tph2 [16]. The mRNA levels of tph2 were determined by quantitative real-time PCR, while its protein levels were evaluated using Western blot analysis and immunohistochemistry.

## Materials and Methods

### Ethics Statement

The experimental procedure was approved by the Indiana University Animal Care and Use Committee (#10525) and was in compliance with the Guiding Principles in the Care and Use of Animals endorsed by the American Physiological Society.

### Animal

**Figure 1:**
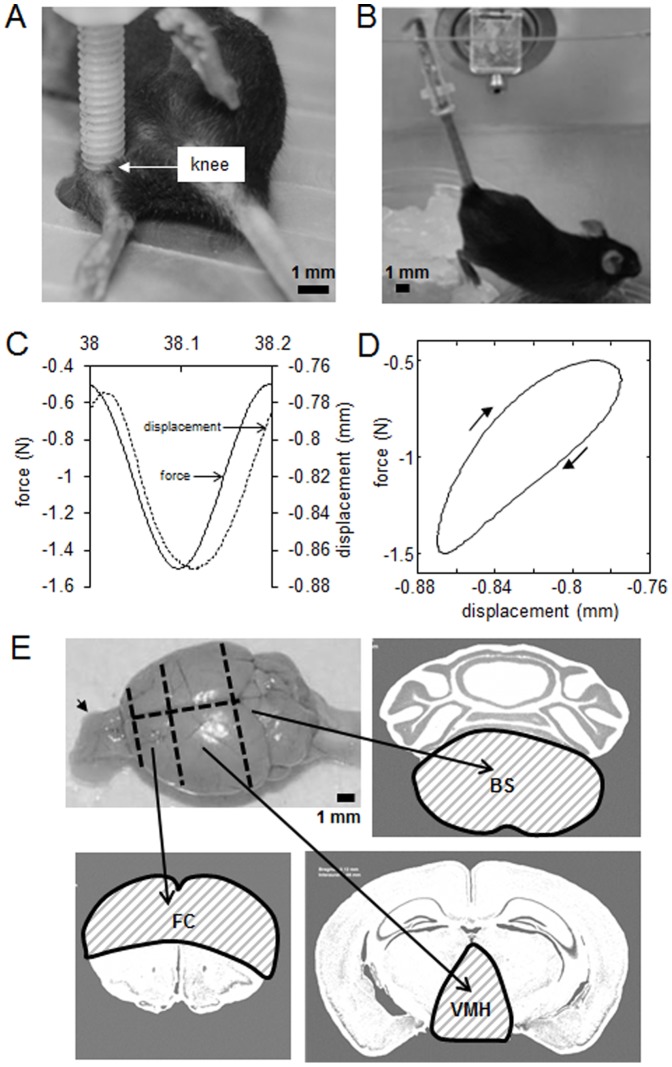
Mechanical treatments and brain samples. (A) Knee loading applied laterally to the mouse knee. (B) Unloading of hindlimbs through tail suspension. (C) Knee loading comprising cyclic compression of 1 N at 5 Hz for 1500 cycles. (D) Force-displacement relationship. (E) Division of a mouse whole brain, containing frontal cortex, FC, ventromedial hypothalamus, VMH, and raphe nuclei of caudal brain stem, BS. The dashed lines indicate approximate split for harvest. The olfactory bulb marked in arrow was excluded. The dissected tissue from three subdivisions is hatched as FC, VMH, and BS.

C57/BL/6 male and female mice, 6 to 8 weeks of age (Harlan Laboratories), were used (n = 72 in total). Two mice were housed per cage, and they were fed with mouse chow and water ad libitum. The animals were allowed to acclimatize for 1 week before the experiment. Animals received knee loading, treadmill exercise, or tail suspension (Fig. 1).

### Knee loading

Cyclic compression was applied to the mouse left knee using an electro-mechanical loading device (ElectroForce® 3100, Bose Corporation, Eden Prairie, MN). The mouse was anesthetized in an anesthetic induction chamber and mask-anesthetized using 2% isoflurane. Lateral loads to the knee were then applied for 5 min at 5 Hz with a peak-to-peak force of 1 N (1,500 cycles). The articular cartilage, femur, tibia, and brain were harvested after the loading bout (n = 21). Internal controls were sham loaded under anesthesia (n = 21).

In response to an applied load (n = 6), the resulting phase shift angle, energy dissipated per cycle, and Young’s modulus were calculated. The phase shift angle between the applied sinusoidal force and displacement of the knee joint was determined using a least square-mean fit method with MATLAB software (version 7.10, The MathWorks, Inc., Natick, MA). The energy loss per cycle was determined as a hysteresis loop integral between the force and displacement waveforms [5]. Young’s modulus was also determined using the linear portion of the force-displacement relationship and dimensions of the loaded section of the knee joint [17].

### Treadmill exercise and tail suspension

Treadmill exercise, mimicking running, was employed as a control of physical activities, while tail suspension was used as a stress inducer. In treadmill exercise, mice (n = 3) were given an acute session of running on a treadmill (5 min at a speed of 12 m/min with 5° inclination per day) for three consecutive days using a commercially available treadmill (Columbus Instruments, Exer 3/6, Columbus, OH) [18]. The treadmill control group (n = 3) was exposed to the same environment as the exercise group, but the treadmill belt was covered with plexiglass to prevent the mouse from touching the surface. In tail suspension, mimicking disuse or unloading of limbs (n = 6), the tail was inserted into a plastic tube of the tail harness. The animal was placed head-down at approximately a 30–40° angle that prohibited the hindlimbs from reaching the ground for 90 min [19]. Animals placed in the same cage in the absence of tail insertion to the tube were used as internal controls (n = 6).

### Quantitative real-time PCR

The previous method was used with modification [17], [20]. Prior to homogenization, femur and tibia were harvested and medullary fluid was washed out. Articular cartilage from distal femur and proximal tibia was pooled for total RNA extraction. For brain, tissues were dissected into three regions including FC, VMH, and BS. The tissues containing dorsal, medial and caudal raphe were labeled as BS (Fig. 1E). The mRNA expressions of each gene were determined using quantitative real-time PCR with the primers listed in Table 1. Expression of collagen types I and II was evaluated for synthesis of bone and cartilage matrix, while NGFß as a neurotrophic factor. As a marker for serotonergic signaling, expression of tph2 was assayed with Sim1 and REST as its potential regulators and Pet 1, Lhx8, and RGS as its downstream effectors [21], [22]. Total RNA was extracted using an RNeasy Lipid Tissue mini kit with QIAzol reagent (Qiagen) and chloroform (Fisher Scientific). Reverse transcription was performed, and real-time PCR was carried out using ABI 7500 with SYBR green PCR kits (Applied Biosystems). The mRNA level of GAPDH was used as an internal control.

**Table 1:** Primer sequence.

Mouse gene	Forward	Backward
Col I	GAGCGGAGAGTACTGGATCG	GCTTCTTTTCCTTGGGGTTC
Col II	GCCAAGACCTGAAACTCTGC	GCCATAGCTGAAGTGGAAGC
NGFß	CCAGTGAAATTAGGCTCCCTG	CCTTGGCAAAACCTTTATTGG
Tph2	CCATCGGAGAATTGAAGCAT	TTCAATGCTCTGCGTGTAGG
REST	GTGCGAACTCACACAGGAGA	AAGAGGTTTAGGCCCGTTGT
Sim1	TGGAAAGCCTCCGAGTCTAA	AGTGAAAGGCGAGGTCAGAA
Pet1	GCACCTCGTTATGACCCCTA	TATACAGGCTGGGGTCCTTG
Lhx8	GGCCTTAGTGTGGCTGAGAG	TGCTCGTCACATACCAGCTC
RGS4	GCTAAGGGGTGAGCACTCTG	TCTGCCCTCACCTAAGCAGT
GAPDH	TGCACCACCAACTGCTTAG	GGATGCAGAGAAGATGTTC

### Western blotting

The previous method was modified and used [17]. Samples isolated from the above regions of brain tissues were dissociated with a mortar and pestle in a RIPA lysis buffer containing inhibitors for proteases and phosphatases (Calbiochem). Isolated proteins were fractionated using 10% SDS gels and electro-transferred to Immobilon-P membranes (Millipore). Immunoblots were carried out using antibodies specific to tph2 (Thermo Scientific) and ß-actin (Sigma). After incubation with secondary antibodies conjugated with HRP, signals were detected with ECL chemiluminescence. Images were captured using an image analyzer (LAS-3000, Fuji Photo Film) and analyzed using Multi Gauge V 3.0 software.

### Immunohistochemistry

Based on the previous method [23], serial coronal sectioning was made to discern dorsal, medial and caudal raphe in the hindbrain or BS. Briefly, glass slides containing cryo-sectioned 9 µm-thick brain tissues were incubated with a primary antibody diluted in blocking solution made of phosphate buffered saline (PBS), Triton X100 and goat serum overnight at 4°C, rinsed, and incubated with the secondary antibody. Primary antibody was rabbit anti-tph2 (1∶200, Thermo Scientific), and secondary antibody was Alexa Fluor® dye-conjugated goat anti-rabbit IgG (diluted 1∶200 in blocking solution, Jackson ImmunoResearch). For nuclear staining, 500 ng/ml of 4′,6-diamidino-2-phenylindole (DAPI, Sigma) was used. Fluorescent images were taken using a BX53 research microscope equipped with a dp72 camera (Olympus). For acquisition of fluorescent micrographs, cell Sens Entry software with built-in scale bar was used (Olympus).

### Statistical analysis

Statistical significance was calculated using Mann–Whitney U test for two group comparisons. For multiple groups, Kruskal–Wallis test followed by Dunn’s *post hoc* test was adopted (PRISM® ver. 3.0). Data are plotted using STATGRAPHICS Centurion® (ver. 16.1.18), and the asterisk (*) denotes *p* < 0.05.

## Results

### Knee loading promoted collagen transcription in bone and cartilage

**Figure 2:**
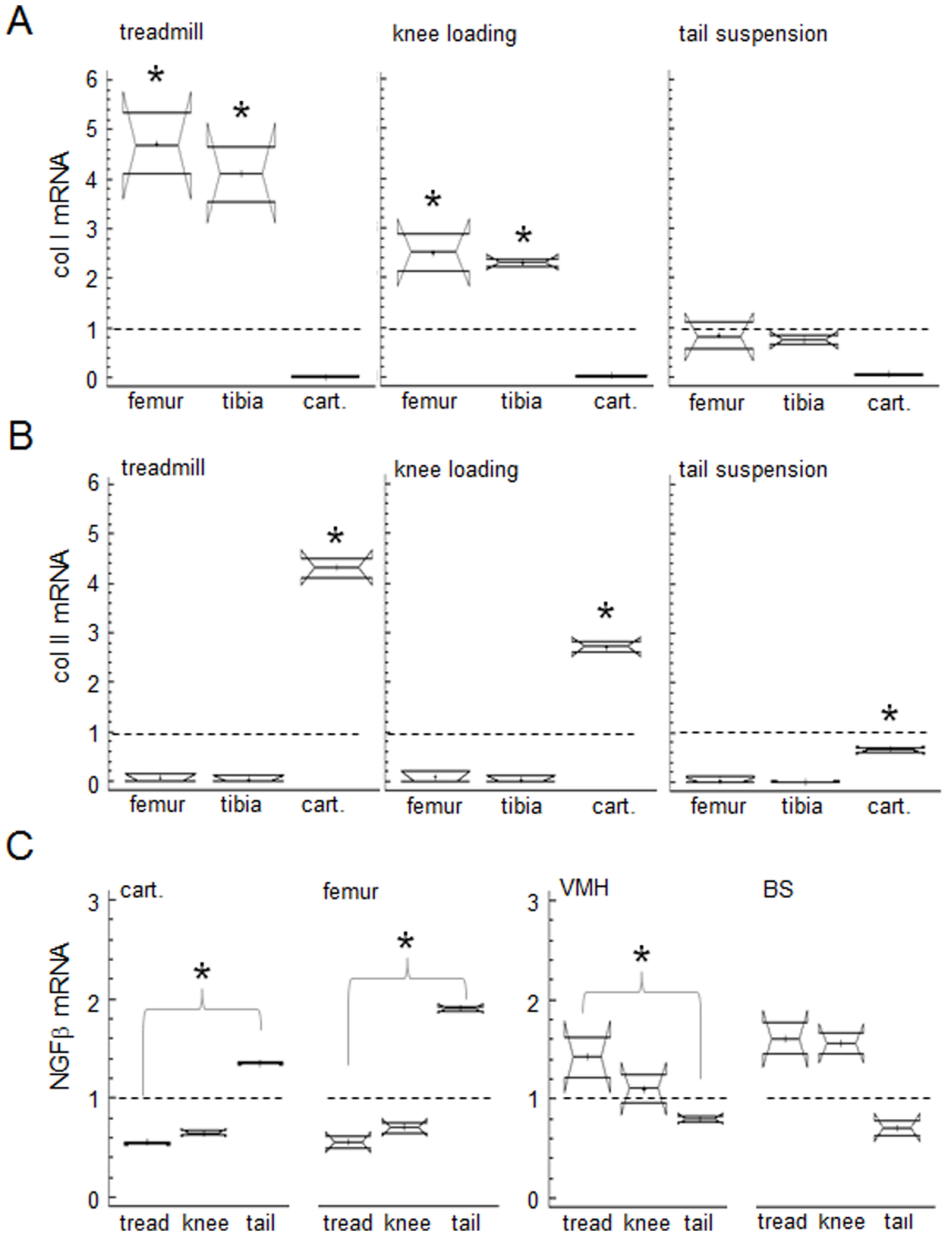
Effects of loading and unloading on mRNA levels of Col I, Col II, and NGFß. (A) Knee loading-induced increases in Col I and unloading-induced decreases in Col I in the femur and tibia. (B) Knee loading-induced increases in Col II and unloading-induced decreases in Col II in cartilage. Dashed lines denote the mRNA levels for untreated controls; A to B. (C) Effects of knee loading on NGFß mRNA. Note that “cart., tread, knee, and tail” denote cartilage, treadmill, knee loading and tail suspension, respectively.

In response to a 1 N force applied at 5 Hz to the mouse knee, the phase shift angle between the force and resulting displacement was found to be 18.1°. The energy loss per cycle was calculated to be 0.201 mJ, and the Young’s modulus of the knee joint was determined as 166 MPa (Fig. 1). Knee loading and treadmill exercise induced significant upregulation of type I collagen mRNA in the femur (p = 0.049) and tibia (p = 0.049), as well as type II collagen mRNA in cartilage (p = 0.049). Furthermore, unloading through tail suspension for 90 min led to significant reduction of type II collagen in cartilage (p = 0.049). However, unloading did not present significant change in type I collagen mRNA as compared to the untreated controls (Fig. 2A-B). Although not significant (p>0.05), knee loading at 1 N showed a decreasing trend of NGFß in the femur and cartilage as compared to the control, consistent with the previous report [12]. In the peripheral joint, tail suspension led to a significant upregulation of NGFß mRNA as compared to the treadmill exercise (Fig. 2 C). Unlike the periphery, tail suspension led to an opposite response of NGFß mRNA in the central nervous system. In the hypothalamic tissue (VMH), tail suspension resulted in a significant downregulation of NGFß mRNA as compared to the treadmill exercise (p<0.05). The plexiglass (n = 3), the sham loaded (n = 3), and the cage control for tail suspension (n = 3) did not show significant difference in mRNA expression of NGFß and collagens as compared to the untreated controls (not shown).

### Knee loading increased tph2 mRNA as compared to unloading

**Figure 3:**
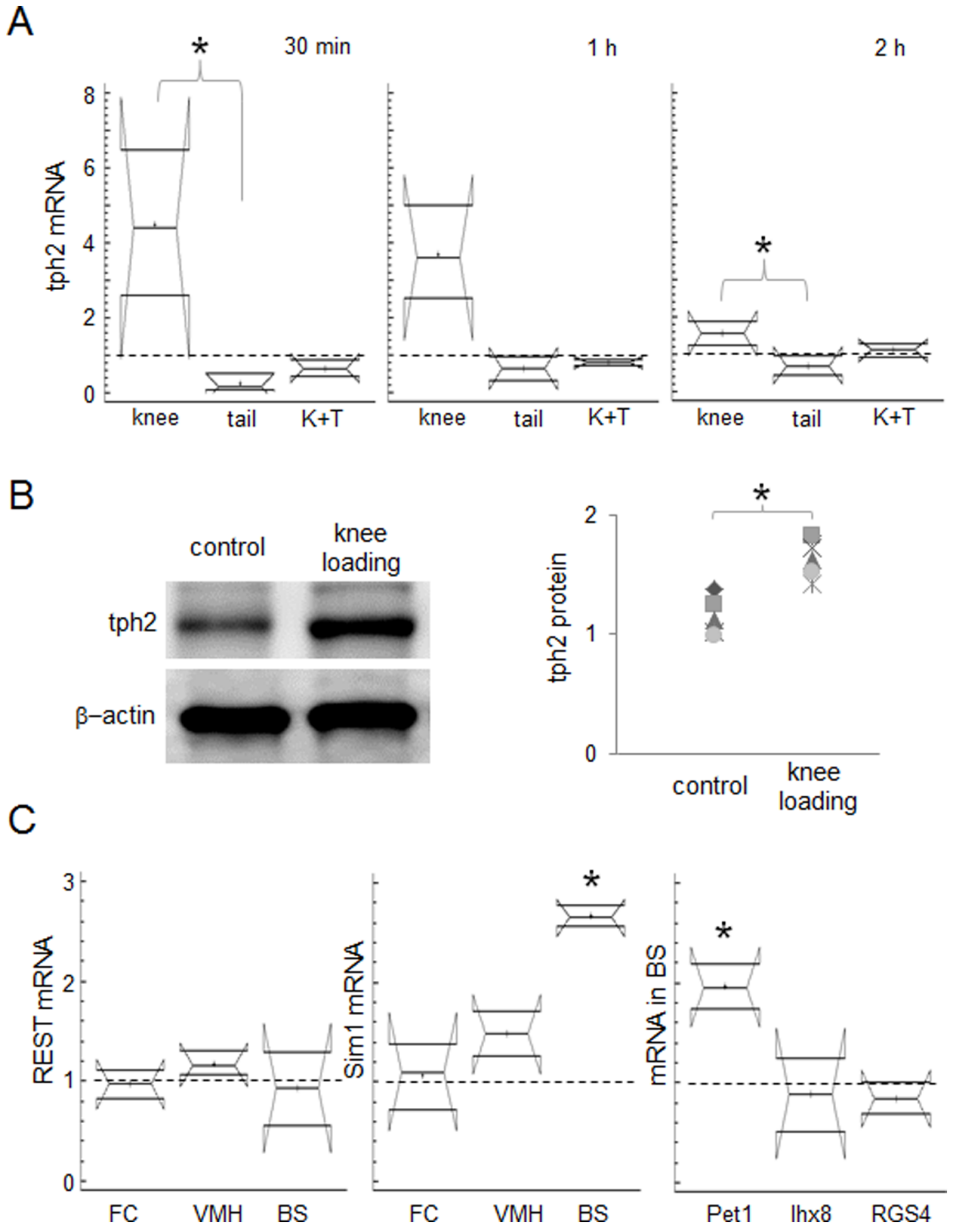
Effects of knee loading on the mRNA and protein levels of tph2 in mouse brain. (A) mRNA levels of tph2 in the BS with knee loading, tail suspension, and knee loading followed by tail suspension. (B) Immunoblots displaying protein levels of tph2 and ß-actin in the BS of mice with knee loading. Next to immunoblots showing tph2 protein fold change. (C) Relative mRNA abundance of REST and Sim1 along with that of Pet 1, lhx8, and RGS4 in the brain in response to knee loading. Dash lines denote the mRNA levels of sham loaded controls. Note that “K+T” denotes knee loading plus tail suspension.

Knee loading led to significant upregulation of tph2 mRNA in the brain or BS, 30 min (p<0.05) following the stimulation of the joint, as compared to the tail suspension. Such load-driven upregulation of tph2 in the BS appeared to persist 1 h following the commencement of mechanical stimulation. At 2 h following the knee loading, mRNA expression of tph2 was significantly higher than that of the tail suspension but to a lesser extent than earlier time points (p<0.05). Over the course of experiments at 30 min, 1 h and 2 h, the combined treatment of knee loading and tail suspension did not show significant change in mRNA expression of tph2 as compared to the control (Fig. 3A). The cage control for tail suspension (n = 3) and the cage control pretreated with sham loading for knee loading plus tail suspension group (n = 3) did not show significant difference in mRNA expression of tph2 as compared to the untreated control (not shown).

### Knee loading increased tph2 protein and upregulated the transcription factor, Sim1

Results on load-induced tph2 mRNA expression in the BS brought us to focus on knee loading. When harvested 2 h following the commencement of mechanical stimulation, knee loading led to a significant increase of tph2 protein in the BS of mice as compared to that of the controls (p = 0.02; Fig. 3B). However, tph2 protein was not detectable in the rostral part of the mouse brain such as FC, regardless of mechanical treatments (data not shown). We further sought to address molecular alterations that may regulate tph2 in the brain. Among two known upstream transcription factors, mRNA expression of Sim1 in the BS (p = 0.01) of mice treated with knee loading was significantly increased as compared to that of the sham loaded controls. Furthermore, mRNA expression of Pet1 [22], was significantly increased in the BS (p = 0.01) with knee loading (Fig. 3C). However, mRNA levels of RE-1 silencing transcription factor, REST, which has been proposed as a transcriptional regulator of tph2 through bipartite neuron-restrictive silencing element in glioma cells was not altered. The mRNA levels of two target genes for tph2, lhx8, and RGS4 were not significantly different in the BS as compared to those of the sham loaded controls. The sham loaded controls (n = 3) did not show significant difference in mRNA expression of REST, Sim1, Pet1, lhx8, and RGS4 as compared to the untreated control (not shown).

### Loading and unloading altered tph2 immunoreactivity in caudal raphe nuclei of the brain

**Figure 4:**
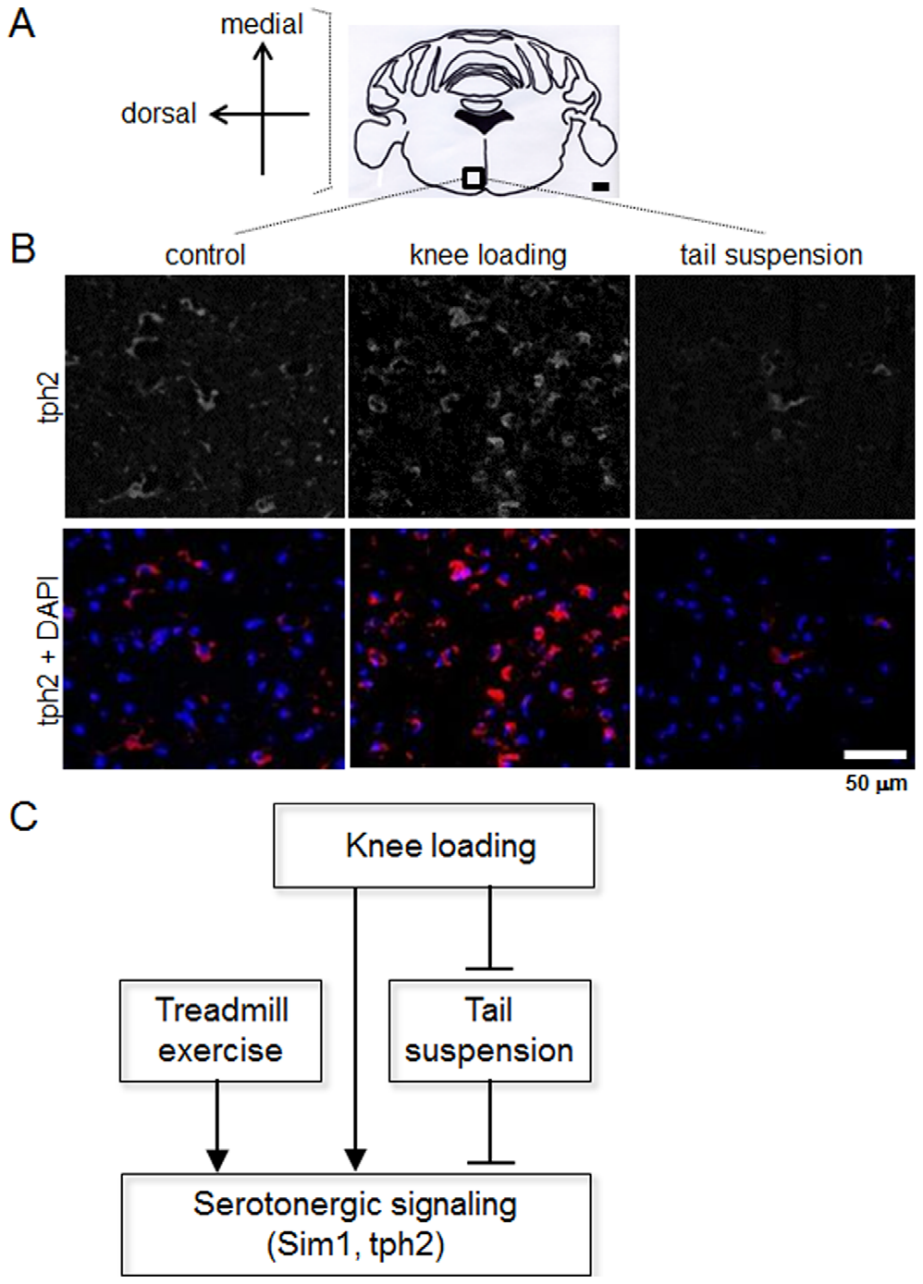
Immunohistochemistry demonstrating the elevated tph2 protein level in the BS with knee loading. (A) Rectangular region of interest in caudal brain sections. Orientation of medial and dorsal corresponds to micrographs in B. (B) Representative micrographs from bregma −5.9±0.1 mm showing tph2 immunoreactivity in mice treated with sham, knee loading, and tail suspension. Nuclear counterstain by DAPI overlaid with tph2 in the lower panels. (C) The proposed role of knee loading and unloading in serotonin synthesis through tph2 in the brain.

While BS showed alteration of tph2 with mechanical stimuli, we sought to identify specific regions that may be associated with load-driven elevation of tph2 in the hindbrain (Fig. 4A). Serial sections containing the dorsal and medial raphe nuclei did not show a significant difference of tph2 immunoreactivity when compared with age-matched controls (data not shown). Adjacent to the midline of caudal raphe nuclei ventral to the fourth ventricle (bregma −5.9±0.1 mm), however, a consistent level of tph2 immunoreactivity was detected in the control animals (Fig. 4A). At the same location, unloading of hindlimbs through tail suspension led to a decrease of tph2 positive staining in caudal raphe, while knee loading induced an increase of tph2 immunoreactivity as compared with the same region in control animals (Fig. 4B).

## Discussion

We presented in this study that physical weight or mechanical loading applied to the knee can elevate tph2 in the hindbrain. Using cyclic loads of 1 N at 5 Hz (1,500 cycles), we demonstrated that knee loading significantly upregulated tph2 mRNA in the BS and the load-driven upregulation of tph2 mRNA persisted at least 2 h. Western blot analyses revealed that tph2 protein was significantly elevated in the BS of mice treated with knee loading. Immunohistochemistry exhibited that knee loading led to an increase of tph2 in raphe nuclei of the BS. Our data provided evidence on tph2 increment in the brain by gentle mechanical loading of the peripheral bone. In response to mechanical loading, we obtained elastic modulus of 166 MPa with energy loss of 0.201 mJ of the mouse knee. These parameters indicate that the loaded tissue is significantly softer and more energy dissipative than bone matrix in the femur and tibia.

Consistent with the load-driven elevation of tph2, treadmill exercise increased its mRNA level and tail suspension decreased it. Immunohistochemistry revealed that the level of tph2 protein in the caudal raphe nuclei of the BS was reduced by tail suspension and elevated by knee loading. We also found that basal level of tph2 mRNA expression and protein secretion in the caudal brain of control animals was consistent and not significantly different among untreated group, sham loaded group, plexiglass group, and cage control for tail suspension, respectively. However, care should be taken in interpretation of our data. Knee loading may not warrant increase of tph2 in the brain because higher magnitude and longer term loading could lead to a stress reaction. Collectively, the results herein support the notion that serotonergic signaling through tph2 might be influenced by mild knee loading for 5 min as a stimulator and by tail suspension for 90 min as an inhibitor (Fig. 4C).

We observed a region-specific localization of tph2, in serial sections of the hindbrain. In response to knee loading, a consistent elevation of tph2 expressing cells was detected in the caudal raphe. In the rostral raphe, tph2 positive cells were detected in all animals but no significant loading effects were observed. It is the raphe nuclei where the brain serotonin system is known to originate, which is numbered B1 to B9. These nine clusters are subdivided into rostral and caudal portion. The caudal portion projects to the spinal cord and cerebellum, which is involved in motor activity, pain, and the regulation of autonomic nervous system including signaling of sympathetic nervous system [16]. Serotonergic neurons in dorsal and medial raphe nucleus project to the forebrain such as cerebral cortex, hippocampus and hypothalamus mediating perception, cognition, and food intake and reproduction [24]. Previously, it is reported that the expression of tph2 is elevated in the dorsal raphe nucleus in depressed suicide [25], [26]. The 28 d voluntary exercise has been shown to induce tph2 in hippocampus, suggesting anti-depressant effects of brain serotonin [27]. Tph2 has been also suggested as a therapeutic target for stress disorders [28]. Yet, in this study, we did not observe a significant alteration of tph2 in the rostral subdivision along with FC and VMH. As the most robust increase in tph2 was in the caudal raphe nuclei that project to the spinal cord and not in the rostral nuclei that project to the forebrain, how our data support behaviorally relevant changes in serotonin after knee loading is in question. It is thought that the threshold for activating tph2 synthesis in the rostral raphe nuclei might be higher, and that whether more sustained mechanical load would evoke a similar upregulation in the forebrain to the hindbrain is the next step to this study.

We also demonstrated molecular changes that may be associated with tph2 in the brain. Knee loading led to increases of transcription factor, Sim 1 and Pet 1 mRNA but not REST/NRSF in the BS [21]. Sim1 has been identified as a regulator of dorsal raphe serotonergic neurons acting upstream of Pet 1 and tph2 [22]. Other target genes of Sim1, lhx8 and RGS4 did not show significant alteration with knee loading applied in the present study. However, we did find the upregulation of lhx8 and RGS4 mRNA in the BS (hindbrain) as compared to the VMH (midbrain; data not shown), as described in the previous report [22]. Further investigation is required to determine the mechanism that underlies the loading-induced tph2 in the brain.

In this study, we showed the effect of peripheral mechanical loading on the brain. The observed remote effect can potentially be mediated through central projecting stimulation and neuronal signaling with neurotrophins. For instance, NGFß is a member of the neurotrophins that is involved in pain sensation and survival of neuron in the brain [12], [29]. Alternatively, loading can be sensed through alterations in the level of hormones and growth factors in the serum. Through signaling molecules in blood circulation, gene expression in the BS might be regulated. Previous studies have shown that knee loading induces direct loading effects, including formation of new bone, stimulation of fracture healing, prevention of cartilage degeneration, and suppression of pain in the knee [5], [6], [7], [12], [17]. The current study indicates that knee loading can modulate brain serotonin level through tph2 in the BS. Further studies are necessary to identify the role of neuronal and endocrine signaling in on-site and remote loading effects.

Although not shown in the present study, insufficient tph2 or lack of serotonin has been shown to lead to mood disorders such as depression [30], schizophrenia [31] and neurodegeneration such as Alzheimer’s dementia [32], [33]. Our mRNA expression study clearly demonstrated that unloading-induced tph2 deficiency is prevented by pre-application of knee loading. Whether mood disorders linked to insufficient tph2 in the brain can be ameliorated by load-induced enhancement of serotonergic signaling will be the next step to investigate.

## Conclusions

We showed that knee loading and treadmill exercises elevated the serotonin producing enzyme, tph2, in the caudal raphe of the hindbrain. Unloading of hindlimbs decreased the mRNA level of tph2, while brief pre-application of knee loading suppressed insufficient tph2 in the brain. An increase in tph2 was associated with an elevation of its transcription factor, Sim 1 and Pet1. It is necessary to evaluate whether the observed upregulation of tph2 increases serotonin transmission in the key action circuit of the brain, which constitute a basis for attenuating mood disorders by physical activities.
